# Supplementary figures and images for: Efficacy and safety of bempedoic acid for the treatment of hypercholesterolemia: A systematic review and meta-analysis
Source: PLoS Med. 2020 Jul 16;17(7):e1003121. doi: 10.1371/journal.pmed.1003121 (PMC7365413; doi:10.1371/journal.pmed.1003121)

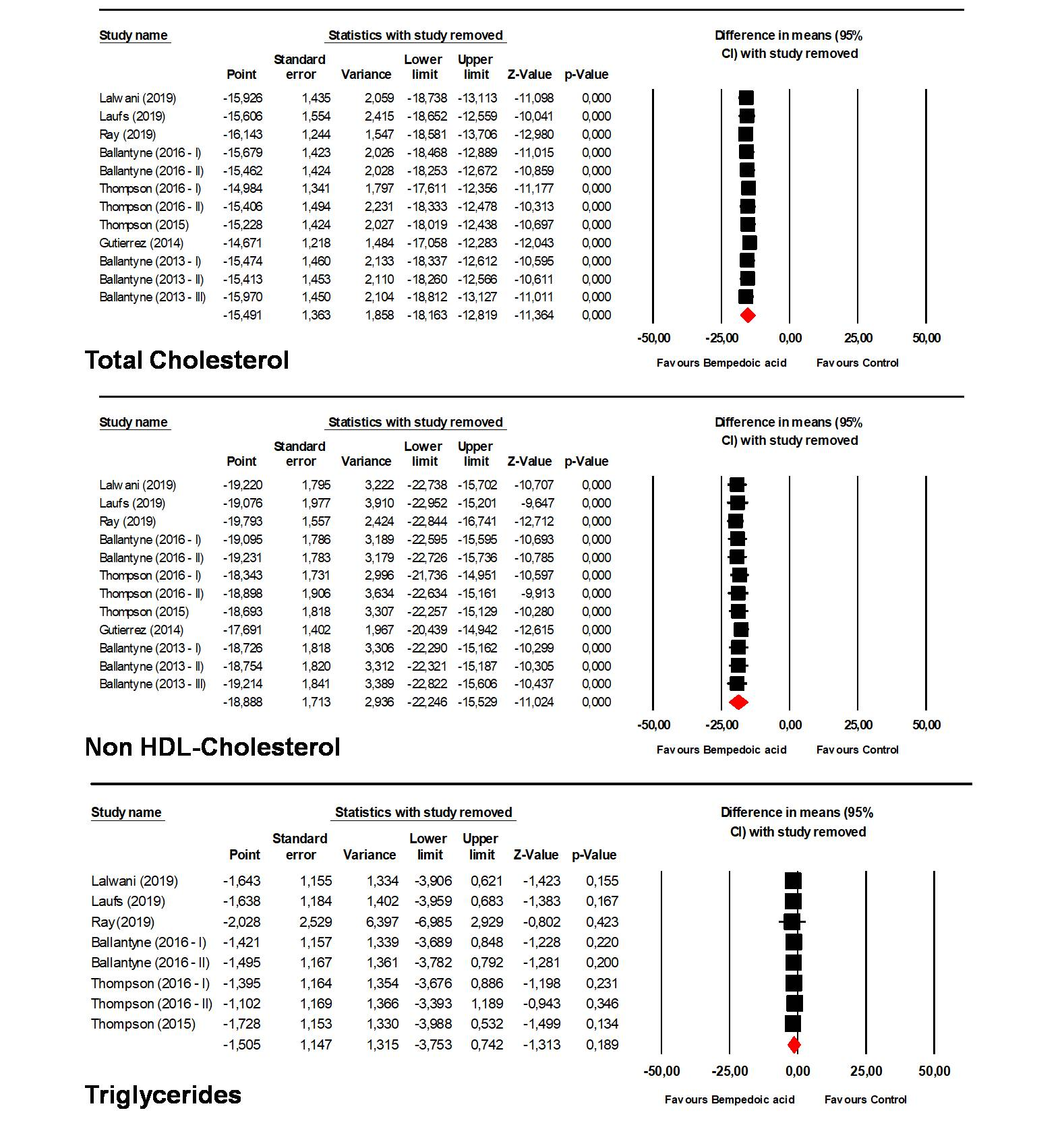

Supplement: S1 Fig — (TIF) [file pmed.1003121.s003.tif]

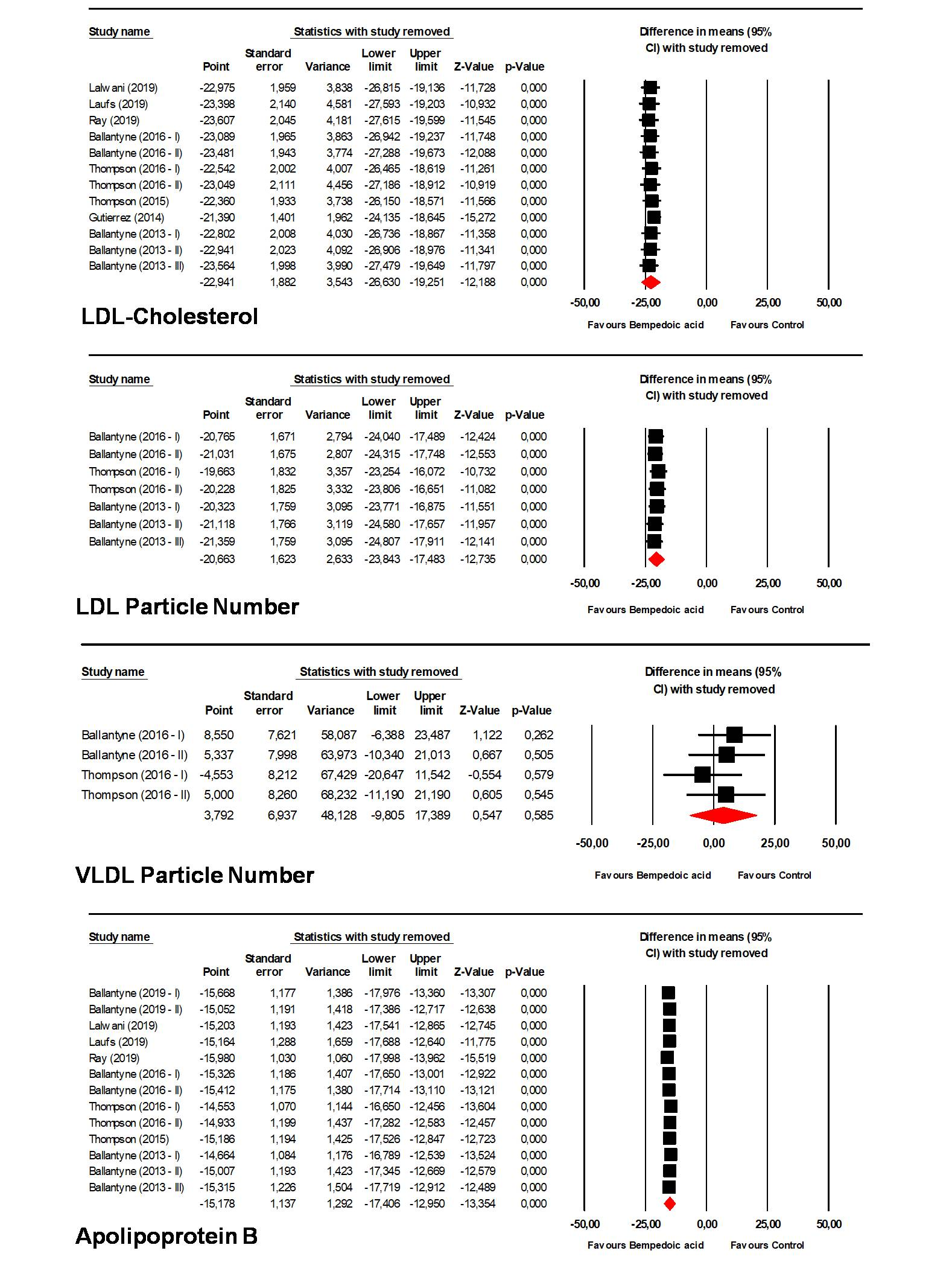

Supplement: S2 Fig — (TIF) [file pmed.1003121.s004.tif]

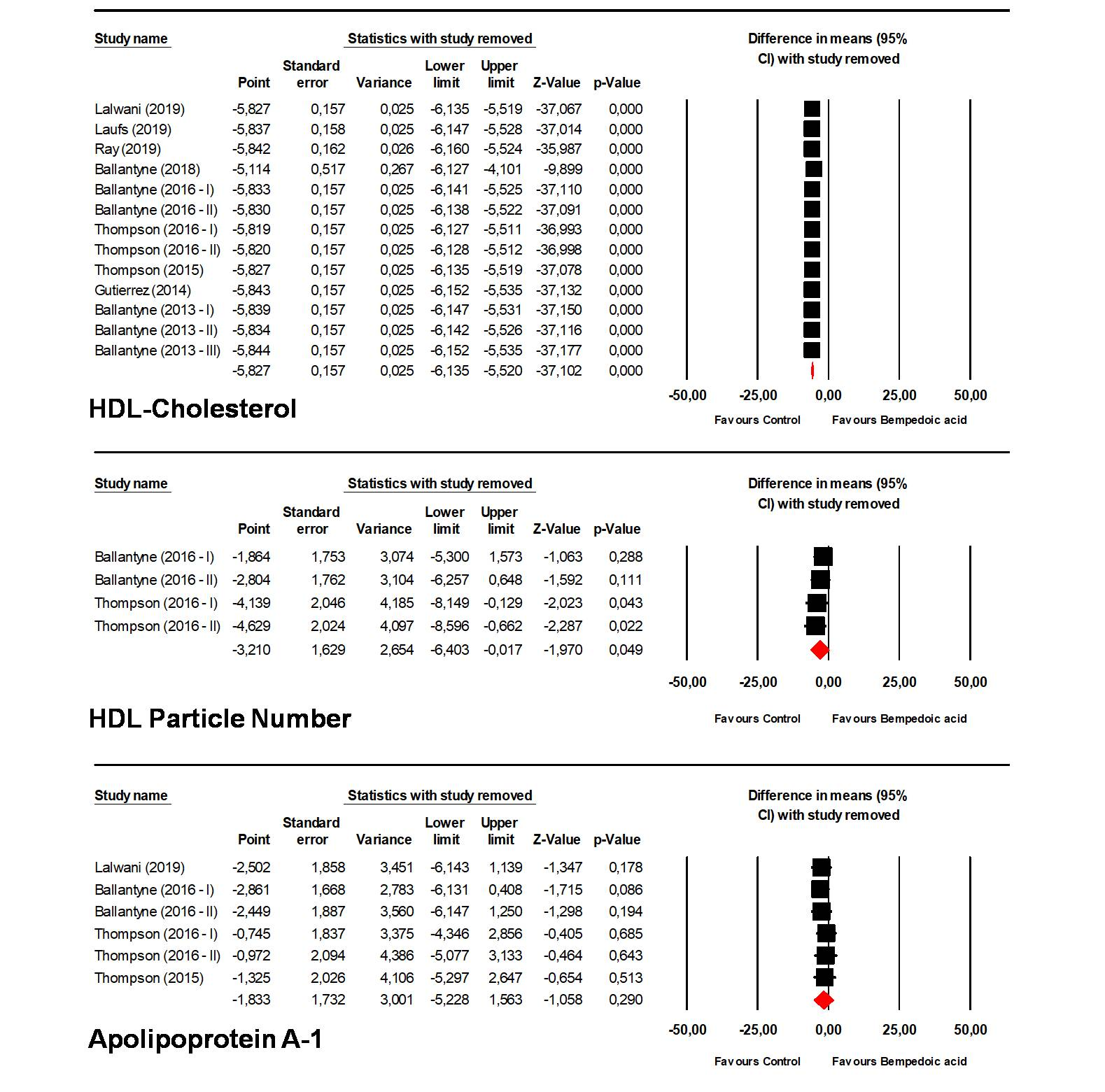

Supplement: S3 Fig — (TIF) [file pmed.1003121.s005.tif]

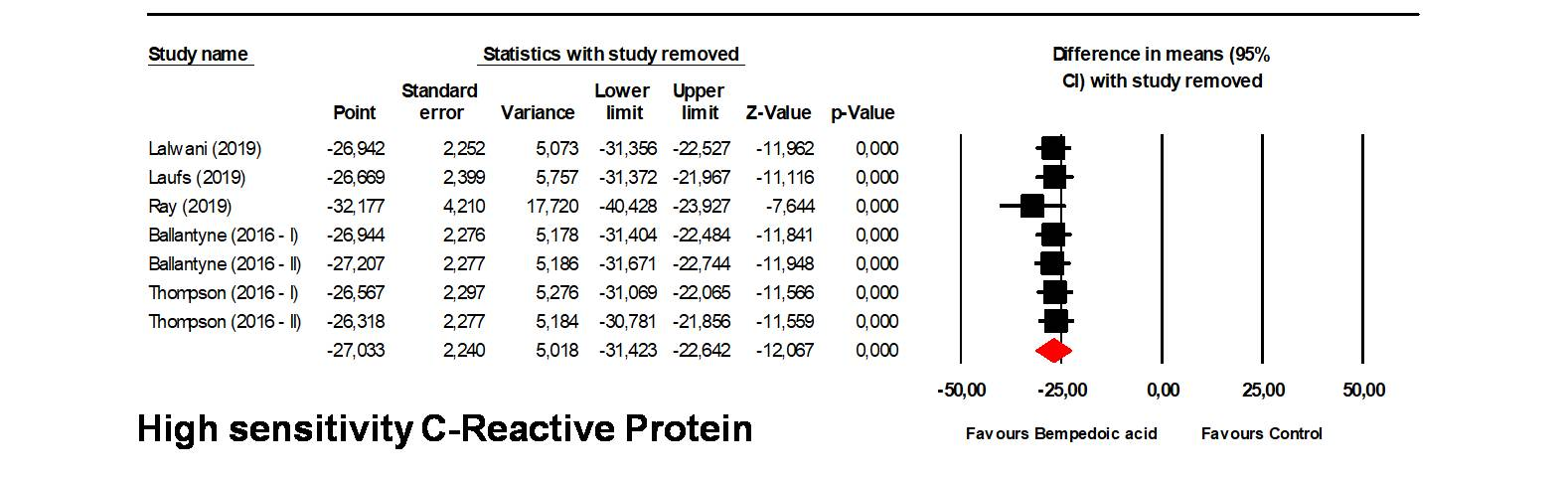

Supplement: S4 Fig — (TIF) [file pmed.1003121.s006.tif]

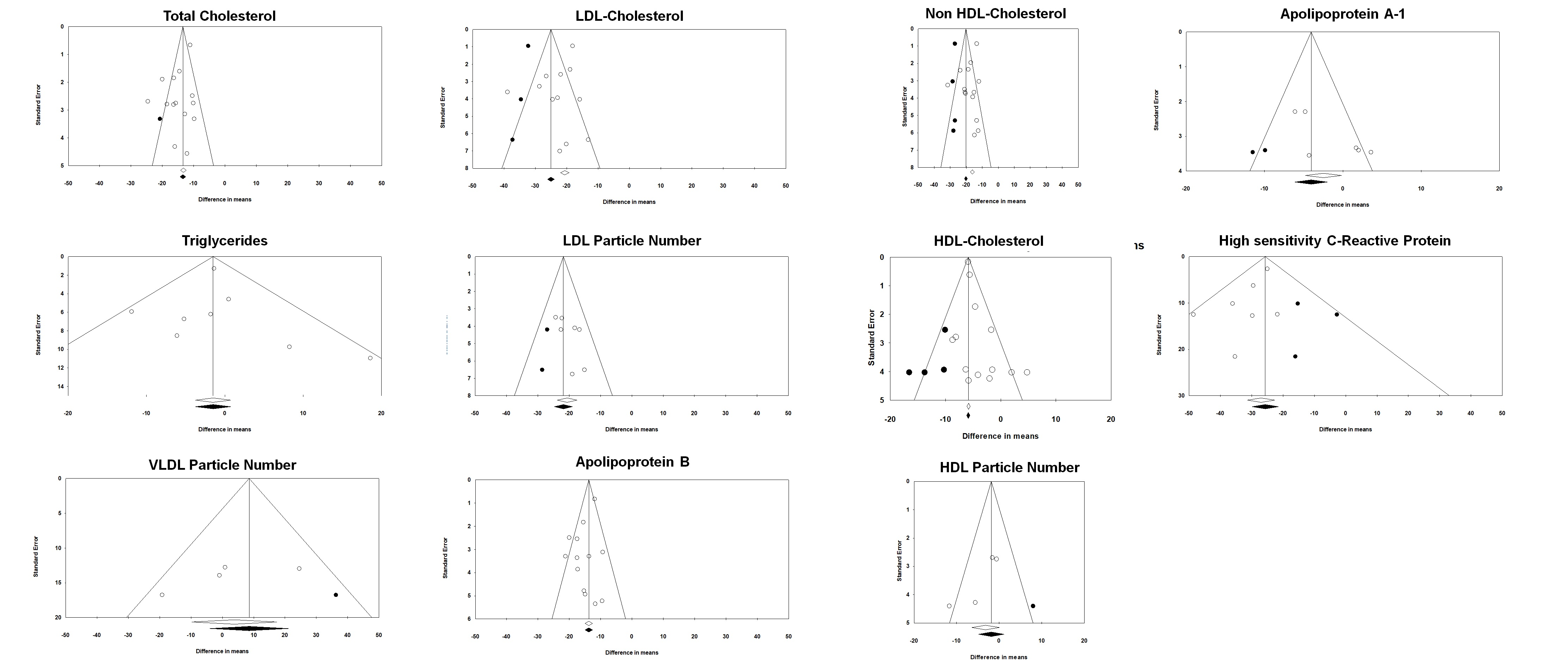

Supplement: S5 Fig — (TIF) [file pmed.1003121.s007.tif]

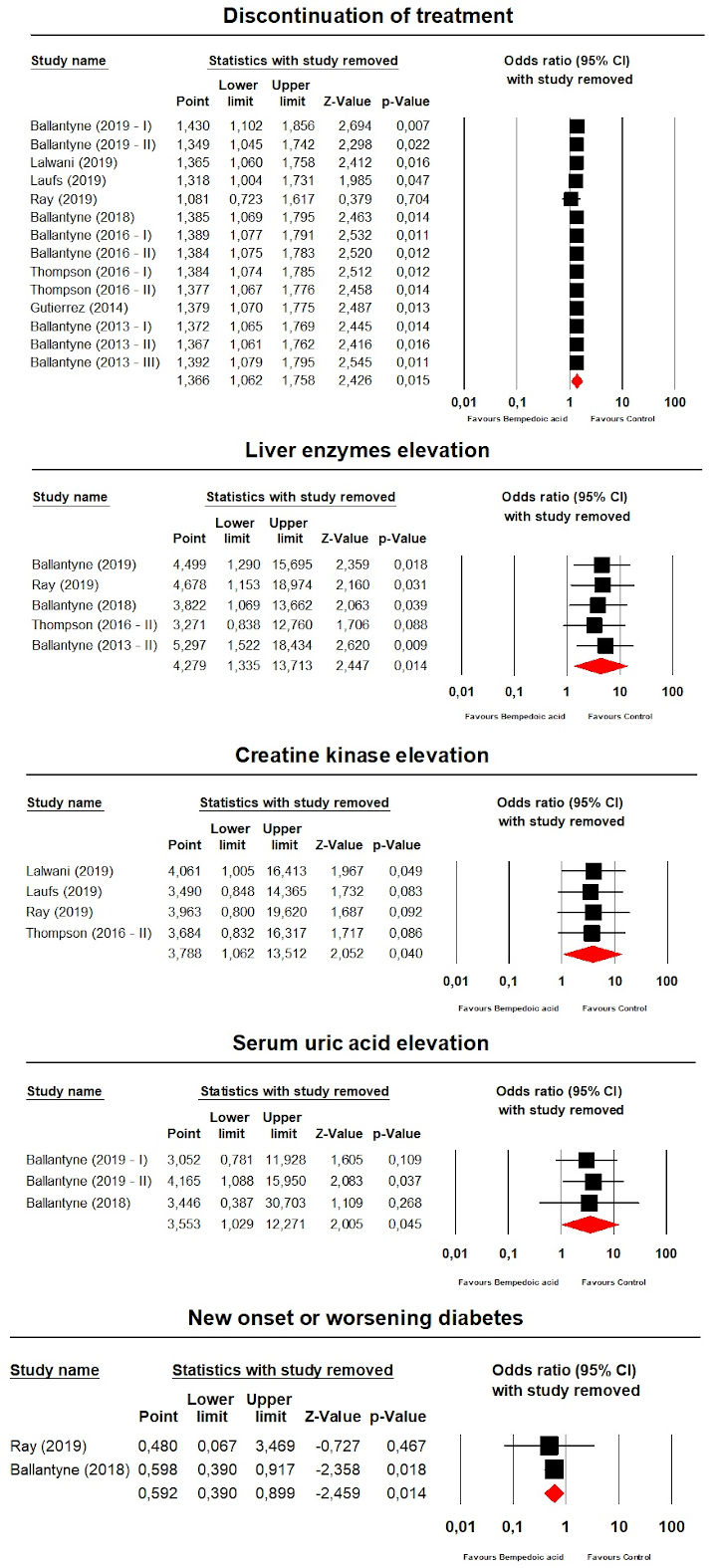

Supplement: S6 Fig — (TIF) [file pmed.1003121.s008.tif]

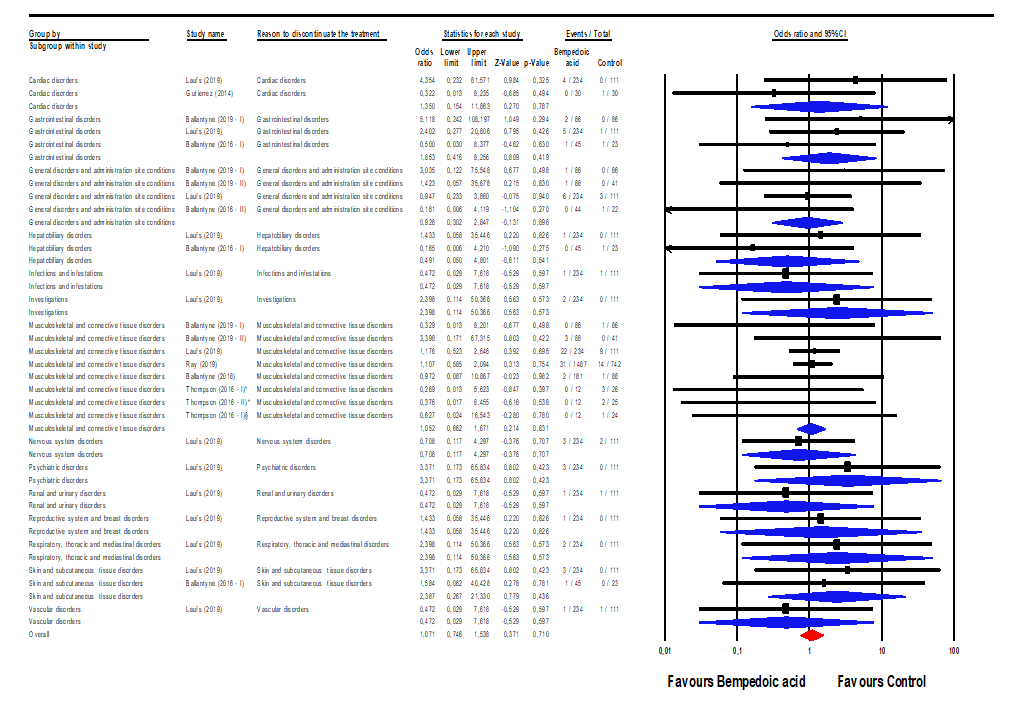

Supplement: S7 Fig — *Data referring to statin-intolerant patients; § Data referring to statin-tolerant patients. (TIF) [file pmed.1003121.s009.tif]

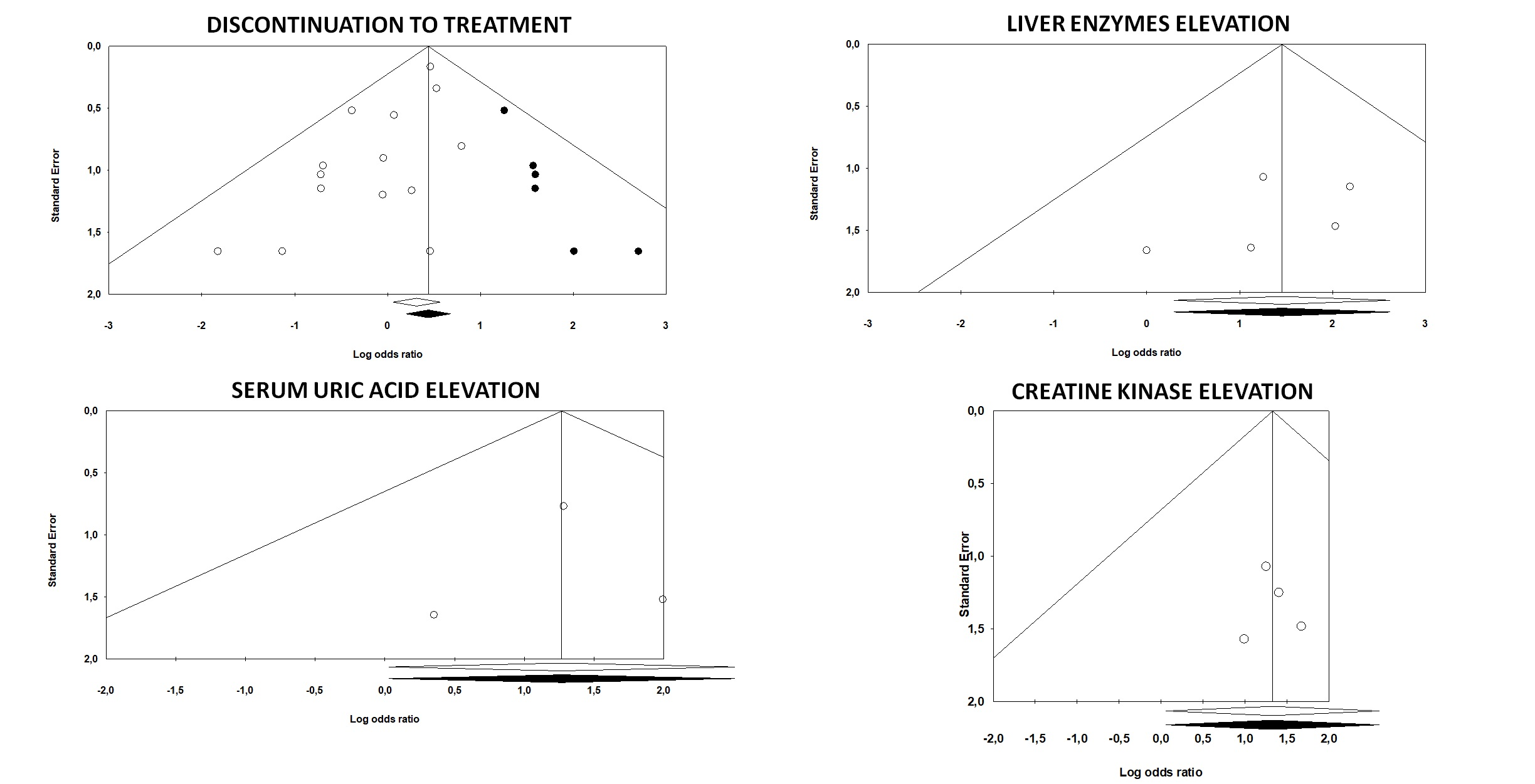

Supplement: S8 Fig — (TIF) [file pmed.1003121.s010.tif]
