# Supplementary material for: Efficacy and safety of bempedoic acid for the treatment of hypercholesterolemia: A systematic review and meta-analysis
Source: PLoS Med. 2020 Jul 16;17(7):e1003121. doi: 10.1371/journal.pmed.1003121 (PMC7365413; doi:10.1371/journal.pmed.1003121)
Supplement: S1 Table — (DOC) [file pmed.1003121.s011.doc]

| ***EFFICACY ANALYSIS*** | | | | | | | | | | | |
| --- | --- | --- | --- | --- | --- | --- | --- | --- | --- | --- | --- |
| Outcome | Differences in mean | | Standard error | Variance | 95% Confidence Interval | | | Z-value | *P*-value | | I^2^ |
|  |  |  |  |  | Lower limit | | Upper limit |  |  |  |  |
| Total Cholesterol | -15·43 | | 1·18 | 1·379 | -17·73 | | -13·12 | -13·133 | <0·001 | | 61·5% |
| Non HDL-Cholesterol | -18·80 | | 1·49 | 2·217 | -21·72 | | -15·88 | -12·6260 | <0·001 | | 60·1% |
| Triglycerides | -2·03 | | 2·53 | 6·397 | -6·99 | | 2·93 | -0·802 | 0·423 | | 26·7% |
| LDL-Cholesterol | -23·69 | | 1·05 | 1·112 | -25·75 | | -24·62 | -22·463 | <0·001 | | 69·7% |
| Apolipoprotein B | -16·05 | | 0·91 | 0·824 | -17·83 | | -14·27 | -17·679 | <0·001 | | 16·4% |
| HDL-Cholesterol | -5·84 | | 0·16 | 0·026 | -6·16 | | -5·52 | -35·987 | <0·001 | | 38·5% |
| High sensitivity C-reactive protein | -32·18 | | 4·21 | 17·720 | -40·43 | | -23·93 | -7·644 | <0·001 | | 0% |
| ***SAFETY ANALYSIS*** | | | | | | | | | | | |
| Outcome | | Odd ratio | | 95% Confidence Interval | | | | Z-value | | *P*-value | I^2^ |
|  |  |  |  | Lower limit | | Upper limit | |  |  |  |  |
| Discontinuation to treatment | | 1·08 | | 0·72 | | 1·62 | | 0·379 | | 0·704 | 0% |
| Liver enzymes elevation | | 4·40 | | 0·69 | | 27·96 | | 1·569 | | 0·117 | 14·8% |
| Creatine kinase elevation | | 3·96 | | 0·80 | | 19·62 | | 1·687 | | 0·092 | 0% |
