# Supplementary material for: Efficacy and safety of bempedoic acid for the treatment of hypercholesterolemia: A systematic review and meta-analysis
Source: PLoS Med. 2020 Jul 16;17(7):e1003121. doi: 10.1371/journal.pmed.1003121 (PMC7365413; doi:10.1371/journal.pmed.1003121)
Supplement: S2 Table — (DOC) [file pmed.1003121.s012.doc]

| ***Begg’s rank correlation*** | | | ***Egger’s linear regression*** | | | | |
| --- | --- | --- | --- | --- | --- | --- | --- |
| Outcome | Tau | *P*-value | Intercept | 95% Confidence Interval | | t-value | *P*-value |
|  |  |  |  | Lower limit | Upper limit |  |  |
| Total Cholesterol | 0·04 | 0·83 | -1·74 | -3·67 | 0·20 | 1·95 | 0·07 |
| Non HDL-Cholesterol | 0·13 | 0·51 | -1·57 | -3·49 | 0·34 | 1·79 | 0·1 |
| Triglycerides | 0·29 | 0·32 | 0·13 | -1·33 | 1·59 | 0·22 | 0·84 |
| LDL-Cholesterol | -0·05 | 0·84 | -1·58 | -3·80 | 0·65 | 1·58 | 0·15 |
| LDL particle number | 0·48 | 0·13 | 1·97 | -0·95 | 4·88 | 1·74 | 0·14 |
| VLDL particle number | -0·67 | 0·17 | -7·93 | -29·02 | 13·17 | 1·62 | 0·25 |
| Apolipoprotein B | 0·22 | 0·3 | -1·11 | -2·51 | 0·30 | 1·73 | 0·11 |
| HDL-Cholesterol | 0·27 | 0·2 | 0·73 | -0·01 | 1·48 | 2·16 | 0·05 |
| HDL particle number | -0·67 | 0·17 | -4·67 | -11·15 | 1·81 | 3·1 | 0·09 |
| Apolipoprotein A1 | 0·6 | 0·09 | 5·25 | -0·27 | 10·76 | 2·64 | 0·06 |
| High sensitivity C-reactive protein | -0·24 | 0·45 | -0·89 | -2·04 | 0·26 | 1·98 | 0·1 |
