# Supplementary material for: Efficacy and safety of bempedoic acid for the treatment of hypercholesterolemia: A systematic review and meta-analysis
Source: PLoS Med. 2020 Jul 16;17(7):e1003121. doi: 10.1371/journal.pmed.1003121 (PMC7365413; doi:10.1371/journal.pmed.1003121)
Supplement: S3 Table — (DOC) [file pmed.1003121.s013.doc]

| Outcome | Z-value for the observed studies | Fail-safe N | *P*-value |
| --- | --- | --- | --- |
|
| Total Cholesterol | -25·084 | 2280 | <0·001 |
| Non HDL-Cholesterol | -23·529 | 2004 | <0·001 |
| LDL-Cholesterol | -25·706 | 2053 | <0·001 |
| LDL particle number | -12·16 | 263 | <0·001 |
| Apolipoprotein B | -19·752 | 1308 | <0·001 |
| HDL-Cholesterol | -15·851 | 838 | <0·001 |
| HDL particle number | -2·389 | 2 | 0·017 |
| High sensitivity C-reactive protein | -10·333 | 188 | <0·001 |
