# Supplementary material for: Efficacy and safety of bempedoic acid for the treatment of hypercholesterolemia: A systematic review and meta-analysis
Source: PLoS Med. 2020 Jul 16;17(7):e1003121. doi: 10.1371/journal.pmed.1003121 (PMC7365413; doi:10.1371/journal.pmed.1003121)
Supplement: S4 Table — AEs = Adverse events. (DOC) [file pmed.1003121.s014.doc]

| Adverse events | Number of studies | Number of treatment arms | Number of adverse events  (active arm / control arm) | Odd ratio | 95% Confidence Interval | | Z-value | *P*-value | I^2^ |
| --- | --- | --- | --- | --- | --- | --- | --- | --- | --- |
|  |  |  |  |  | Lower limit | Upper limit |  |  |  |
| Any AEs | 10 | 15 | 1743/908 | 1·07 | 0·92 | 1·25 | 0·889 | 0·374 | 37·4% |
| Serious AEs | 8 | 11 | 253/126 | 1·04 | 0·83 | 1·31 | 0·336 | 0·737 | 0% |
| Study drug related AEs | 8 | 13 | 196/90 | 1·29 | 0·96 | 1·74 | 1·702 | 0·089 | 11·5% |
| Major adverse cardiac events | 2 | 3 | 71/45 | 0·79 | 0·54 | 1·17 | -1·180 | 0·238 | 0% |
| Muscle-related AEs | 9 | 14 | 277/126 | 1·15 | 0·91 | 1·44 | 1·189 | 0·234 | 0% |
| Arthralgia | 7 | 12 | 30/24 | 0·71 | 0·39 | 1·28 | -1·131 | 0·258 | 0% |
| Gout | 3 | 3 | 22/4 | 2·39 | 0·76 | 7·46 | 1·493 | 0·135 | 41% |
| Hypertension | 2 | 3 | 14/4 | 1·75 | 0·53 | 5·75 | 0·923 | 0·356 | 0% |
| Back pain | 4 | 6 | 13/16 | 0·65 | 0·30 | 1·40 | -1·106 | 0·269 | 0% |
| Rash | 2 | 3 | 3/1 | 0·92 | 0·15 | 5·78 | -0·090 | 0·928 | 0% |
| Headache | 8 | 12 | 44/17 | 1·46 | 0·80 | 2·65 | 1·240 | 0·215 | 0% |
| Fatigue | 4 | 7 | 20/10 | 0·86 | 0·41 | 1·83 | -0·392 | 0·695 | 0% |
| Dizziness | 4 | 5 | 9/4 | 0·72 | 0·18 | 2·86 | -0·473 | 0·636 | 28·4% |
| Abdominal pain | 2 | 2 | 0/4 | 0·14 | 0·02 | 1·31 | -1·721 | 0·085 | 0% |
| Nausea | 5 | 8 | 20/6 | 1·16 | 0·44 | 3·05 | 0·304 | 0·761 | 0% |
| Constipation | 4 | 5 | 9/5 | 1·63 | 0·54 | 4·89 | 0·872 | 0·383 | 0% |
| Diarrhea | 2 | 4 | 9/3 | 0·94 | 0·26 | 3·42 | -0·101 | 0·919 | 0% |
| Naso-pharyngitis | 4 | 6 | 18/9 | 1·54 | 0·64 | 3·74 | 0·960 | 0·337 | 0% |
| Upper respiratory tract infection | 5 | 6 | 11/7 | 0·99 | 0·34 | 2·88 | -0·014 | 0·989 | 15·8% |
| Bronchitis | 4 | 5 | 13/11 | 0·60 | 0·27 | 1·37 | -1·213 | 0·225 | 0% |
| Urinary tract infection | 7 | 11 | 35/26 | 0·75 | 0·43 | 1·31 | -1·003 | 0·316 | 0% |
| Decrease in glomerular filtration rate | 2 | 2 | 12/0 | 6·24 | 0·81 | 48·26 | 1·755 | 0·079 | 0% |
| Increase in creatinine level | 2 | 3 | 16/3 | 2·30 | 0·77 | 6·88 | 1·486 | 0·137 | 0% |
