# Supplementary material for: Efficacy and safety of bempedoic acid for the treatment of hypercholesterolemia: A systematic review and meta-analysis
Source: PLoS Med. 2020 Jul 16;17(7):e1003121. doi: 10.1371/journal.pmed.1003121 (PMC7365413; doi:10.1371/journal.pmed.1003121)
Supplement: S5 Table — (DOC) [file pmed.1003121.s015.doc]

| Study | Reason to discontinuation | Percentage of discontinuation | |
| --- | --- | --- | --- |
|  |  | Bempedoic acid | Control |
| Ballantyne (2019 - I)^22^ | Musculoskeletal and connective tissue disorders | 0% | 1·2% |
|  | General disorders and administration site conditions | 1·2% | 0% |
|  | Gastrointestinal disorders | 2·3% | 0% |
| Ballantyne (2019 - II) ^22^ | Musculoskeletal and connective tissue disorders | 3·4% | 0% |
|  | General disorders and administration site conditions | 1·1% | 0% |
| Laufs (2019)^9^ | Musculoskeletal and connective tissue disorders | 9·4% | 8·1% |
|  | General disorders and administration site conditions | 2·6% | 2·7% |
|  | Gastrointestinal disorders | 2·1% | 0·9% |
|  | Nervous system disorders | 1·3% | 1·8% |
|  | Cardiac disorders | 1·7% | 0·0% |
|  | Psychiatric disorders | 1·3% | 0·0% |
|  | Skin and subcutaneous tissue disorders | 1·3% | 0·0% |
|  | Investigations | 0·9% | 0·0% |
|  | Respiratory· thoracic and mediastinal disorders | 0·9% | 0·0% |
|  | Infections and infestations | 0·4% | 0·9% |
|  | Renal and urinary disorders | 0·4% | 0·9% |
|  | Vascular disorders | 0·4% | 0·9% |
|  | Hepatobiliary disorders | 0·4% | 0·0% |
|  | Reproductive system and breast disorders | 0·4% | 0·0% |
| Ray (2019) ^23^ | Musculoskeletal and connective tissue disorders | 2·1% | 1·9% |
| Ballantyne (2018) ^24^ | Musculoskeletal and connective tissue disorders | 1·1% | 1·1% |
| Ballantyne (2016 - I) ^25^ | Hepatobiliary disorders | 0·0% | 4·3% |
|  | Gastrointestinal disorders | 2·2% | 4·3% |
|  | Skin and subcutaneous tissue disorders | 2·2% | 0·0% |
| Ballantyne (2016 - II) ^25^ | General disorders and administration site conditions | 0·0% | 4·5% |
| Thompson (2016 - I)* ^26^ | Musculoskeletal and connective tissue disorders | 0·0% | 11·5% |
| Thompson (2016 - I)^§ 26^ | Musculoskeletal and connective tissue disorders | 0·0% | 4·2% |
| Thompson (2016 - II)* ^26^ | Musculoskeletal and connective tissue disorders | 0·0% | 8·0% |
| Gutierrez (2014) ^27^ | Cardiac disorders | 0·0% | 3·3% |

* Data referring to statin-intolerant patients·

^§^ Data referring to statin-tolerant patients·
