# Supplementary material for: Efficacy and safety of bempedoic acid for the treatment of hypercholesterolemia: A systematic review and meta-analysis
Source: PLoS Med. 2020 Jul 16;17(7):e1003121. doi: 10.1371/journal.pmed.1003121 (PMC7365413; doi:10.1371/journal.pmed.1003121)
Supplement: S6 Table — (DOC) [file pmed.1003121.s016.doc]

| Outcome | Z-value for the observed studies | Fail-safe N | *P*-value |
| --- | --- | --- | --- |
|
| Creatine kinase elevation | 2·031 | 1 | 0·042 |
| Transaminase elevation | 2·302 | 2 | 0·021 |
